# Pain Science Education for People With Persistent Pain on NHS Waiting Lists: A Mixed Methods Study

**DOI:** 10.1155/prm/9944170

**Published:** 2026-01-20

**Authors:** Mankelow J., Ryan C. G., Stanton T. R., Pell R., Varghese V., Martin D.

**Affiliations:** ^1^ School of Health and Life Sciences, Teesside University, Middlesbrough, UK, tees.ac.uk; ^2^ Pain Education Team to Advance Learning (PETAL) Collaboration, Adelaide, Australia, tees.ac.uk; ^3^ IIMPACT in Health, Allied Health and Human Performance, The University of South Australia, Adelaide, South Australia, Australia, unisa.edu.au; ^4^ Persistent Pain Research Group, Hopwood Centre for Neurobiology, Lifelong Health Theme, South Australian Health and Medical Research Institute (SAHMRI), Adelaide, South Australia, Australia; ^5^ Flippin’ Pain Campaign Director, Cora Health, Newcastle upon Tyne, UK; ^6^ NIHR Applied Research Collaboration, NIHR Applied Research Collaboration for the North East and North Cumbria, Newcastle upon Tyne, UK

**Keywords:** pain science education, waiting list, webinars

## Abstract

**Background:**

Persistent pain is a leading cause of disability worldwide. Long waiting times can adversely affect outcomes and impede effective management; thus, waiting list‐targeted interventions may be beneficial.

**Objective:**

To explore the perceptions of people with persistent pain receiving pain science education (PSE) online, en masse, whilst on an NHS waiting list.

**Methods:**

A mixed‐methods observational study of people with persistent pain on NHS waiting lists was undertaken. PSE‐informed webinars were delivered by *Flippin’ Pain,* a U.K.‐based public health campaign. A questionnaire was circulated postwebinar exploring participants’ experience of the webinars collectively and future behavioural intentions. Quantitative data were analysed using descriptive statistics. Reflexive thematic analysis was used to analyse qualitative data.

**Results:**

Participants’ (*n* = 114) pain originated primarily from musculoskeletal sources. Postwebinar, participants felt more hopeful about their future (64%), intended to increase activity levels (71%), intended to reduce their opioid use (51%) and felt that they may be more confident to talk to others about their pain (42%). Overall the webinars were well received; they were considered interesting, and participants reported that they provided feelings of hope and empowerment. 80% of participants would recommend the webinars to others, although a small minority of participants were strongly negative about the webinars and found them unhelpful.

**Conclusion:**

Participants on NHS waiting lists with persistent pain predominantly found PSE webinars helpful. It shifted their understanding of pain, and they intended to undertake self‐management activities in line with evidence‐based care. Appropriately powered RCTs are warranted to robustly investigate the effectiveness of en masse PSE for patients awaiting care.

## 1. Introduction

Persistent pain is defined as pain that persists for 3 months or more [1]. It is the leading cause of disability, globally affecting one in four adults [2, 3], and is associated with high personal and socio‐economic costs [4, 5]. Waiting lists for any condition numbered 7.5 million in the United Kingdom in 2024 [6]. These lists include general surgery, urology, rheumatology, trauma and orthopaedics, ear, nose and throat, neurosurgery, cardiothoracic surgery, mental health and paediatric services. Pain is a common feature of the many different waiting lists. Prolonged waits are associated with poorer outcomes for people with pain [7–9]. Those from areas with greater health inequity are at an increased risk of developing complications whilst waiting and experience worse health outcomes [10].

For those with persistent pain, waiting times for pain management services in Britain vary from 6 weeks to 2.2 years [11]. Waiting times of ≥ 6 months for pain management are associated with deterioration in health‐related quality of life and an increase in depression scores [9]. In contrast, there is an association between early access to musculoskeletal services and improvement in patient outcomes, including work participation [12].

Many patients (82%) awaiting care note the absence of information relative to their health condition whilst they wait [13]. Those with lower incomes are more likely to report that they did not receive information during the waiting period and/or that they found information received ‘inaccessible’ or ‘of limited use’ [13]. Worryingly, some report a sense of ‘abandonment’ during which they lost hope. While hope has multifactorial contributors, the presence of hope shows important and positive impacts on persistent pain [14]. Hopefulness in the presence of persistent pain is associated with lower levels of anxiety and depression and a greater level of self‐efficacy [15], the latter being important for successful self‐management, a goal iterated by numerous pain guidelines.

Lack of information can mean that those who wish to self‐manage may not know how or where to start. The absence of credible information may also mean that common misconceptions about persistent pain, commonly rooted in an outdated structural pathology model [16], go unchecked and hinder effective self‐management. These misconceptions increase patient expectations for traditional passive treatments, which are of low value and can result in disappointment and frustration if they are subsequently not received [17]. Contrastingly, when offered evidence‐based active physical and psychological therapies, patients can often understandably reject these treatments and feel unheard or invalidated if offered them [18].

Pain science education (PSE) is an intervention that aims to address structural pathology based misconceptions about pain by helping people reconceptualise pain, shifting its meaning from a marker of tissue injury/damage to that of a marker of the perceived need to protect the body from real or potential danger [19]. This educational approach is in keeping with contemporary pain management guidelines [20, 21] and has been shown to improve pain knowledge, reduce movement evoked pain, alleviate worry about tissue damage, as well as reduce pain levels and associated disability [22–25].

PSE is typically delivered in a one‐to‐one or small group setting to promote individualisation. However, its role in public health education is growing [18]. Early educational intervention for waiting list patients may reduce pain‐related worry and encourage positive (and recommended) lifestyle behaviours prior to their appointment. Additionally, it may mean that expectations of care shift towards evidence‐based, biopsychosocial interventions and away from non‐evidence‐based interventions that hold potential iatrogenic harm and waste limited resources [26]. A systematic review found that group interventions before individual pain clinic appointments can improve service delivery, waiting times and cost per patient [27]. However, there is some conflict within the literature, with other studies finding no benefit of brief preclinical education on function for people with persistent pain [28]. Further research is warranted to better understand the impact of PSE delivered as part of a public health approach to persistent pain.

This study aimed to explore the perceptions of people with persistent pain of receiving PSE online en masse whilst on an NHS waiting list.

## 2. Methodology

### 2.1. Study Design

This was a mixed‐methods, observational study. Descriptive evaluation of quantitative data and reflexive thematic analysis of qualitative data enabled a detailed exploration of the processes through which participants made sense of the information presented [29]. Ethical approval was obtained from Teesside University (Application: 11120). The **ST**rengthening the **R**eporting of **Ob**servational Studies in **E**pidemiology (STROBE) guidelines [30] and the Consolidated Criteria for Reporting Qualitative Research (COREQ) were used to guide reporting [31].

### 2.2. Participants and Recruitment

Adult patients with persistent pain (duration ≥ 3 months) from any NHS clinical waiting list for the management of their pain were eligible to take part. Potential participants were provided with a participant information sheet and invited to complete an anonymised questionnaire. Participants were recruited via a variety of different methods, including social media. As part of the advertisement for the webinars, healthcare professionals were invited to send the link to the events to individuals on their waiting lists. There were no exclusion criteria for attending the webinars; they were freely available to the public but tailored for waiting list individuals with pain.

### 2.3. PSE Intervention

The PSE intervention of three webinars was delivered as part of a public health campaign, *Flippin’ Pain* (https://www.flippinpain.co.uk), which is a U.K.‐based initiative to improve public understanding of pain. The intervention consisted of three online webinars which were run in February and March 2022. The webinars (Table 1) were presented by healthcare professionals with several years’ experience of delivering PSE to a variety of different audiences. Each webinar lasted ∼90 min and consisted of a 45–60 min didactic presentation followed by a Q&A with a panel of clinicians and people with lived experience of pain. The webinars included key intervention components of the Behaviour Change Wheel [32], including education, modelling (by individuals with lived experience), enablement and a level of training. There was a running theme in the messaging of the three webinars relating to the biopsychosocial nature of persistent pain, but with the focus shifting slightly to ensure relevance for a breadth of participants. Thus, the three webinars have been assessed as one entity.

**Table 1:** Presentations in the pain science education webinar series.

Number in series, date, presenter, title	Aim
Webinar one: https://www.youtube.com/watch?v=TO_3smvAjaY Tues 15th Feb, Prof. Lorimer Moseley ‘Rethinking Pain: New Understanding & New Possibilities’	Address bioplasticity, PSE and the potential to change.

Webinar two: https://www.youtube.com/watch?v=xFkIgFksRQo Wed 2nd Mar, Prof. Cormac Ryan ‘Why Everything Matters When it Comes to Pain’	Communicate two key messages of the Flippin’ Pain campaign: ‘hurt does not always mean harm’ and ‘everything matters when it comes to pain’. Illustrated how evidence‐based active physical and psychological therapies purport to work so as to encourage attendees to opt for these strategies as part of their pain management.

Webinar three: https://www.youtube.com/watch?v=oHn0F1CU8GQ. Wed 23rd Mar, A/Prof. Tasha Stanton ‘Flippin’ Everything You Thought You Knew About Arthritis’	Target misconceptions about arthritis by increasing knowledge of the contemporary understanding of arthritis (and thus why education, physical activity and inflammation management are so important) to provide hope for improvement. and involving people with lived experience of persistent pain who had been able to use this information.

Webinar transcripts were evaluated for readability and understandability of the content using the Hemingway App (https://hemingwayapp.com/readability-checker). Readability and understandability of the transcripts were scored ‘good’ (scoring six or seven out of 10 over the three webinars) when assessed with the Hemingway App. These scores suggest that the readability of the transcript would be accessible to a reader in the sixth or seventh grade, which is the average reading age in the United Kingdom (11–14 years) (NHS Health Education England, https://library.nhs.uk/wp-content/uploads/sites/4/2020/08/Health-literacy-how-to-guide.pdf, [33]).

Immediately after attending the webinar, those who were willing to participate in the study were asked to complete an online questionnaire. The questionnaire (Table 2) featured a mix of 17 quantitative and qualitative questions. Questions specifically focused on the following: the webinar’s effect on hopefulness; intention to undertake evidence‐supported behaviours/treatments (physical activity); intention to undertake non‐evidence‐based behaviours/treatments (scans, opioids and surgery); whether and how they would recommend the webinars; and what they found useful. A number of these questions related to key components of the Behaviour Change Wheel [32], such as credibility of the intervention (would recommend to others) and coherence with guideline‐based treatment recommendations.

**Table 2:** Questionnaire to evaluate the PSE webinar series.

No.	Question	Possible responses
1	Please select all the events in this webinar series that you have watched. Please don′t select events that are in the future, even if you are intending to watch them.	Tues 15th Feb, ‘Rethinking Pain: New Understanding & New Possibilities’ with Prof. Lorimer Moseley Wed 2nd Mar, ‘Why Everything Matters when it comes to Pain’ with Prof. Cormac Ryan Wed 23rd Mar, ‘Flippin’ Everything You Thought You Knew About Arthritis’ with A/Prof. Tasha Stanton

2	Do you live with chronic pain? (Chronic pain is defined as pain that has persisted longer than 3 months)	Yes No

3	Are you currently waiting for an appointment or treatment with an NHS service?	Yes No

4	If yes, which of the following best describes the service you are waiting for?	Orthopaedics (T&O) Pain management service/pain clinic Rheumatology Physiotherapy/musculoskeletal (MSK) Other

5	If other, please tell us what service you are waiting for	Free text

6	Are you a healthcare professional?	Yes No

7	If not a healthcare professional: Physical activity ‘Having watched the webinar(s)…	I plan to try and increase the amount of physical activity I do I plan to seek advice on how to safely increase the amount of physical activity I do I don’t plan on making any changes to the amount of physical activity I do I plan to reduce the amount of physical activity I do

8	Scans Having watched the webinar(s), if given the option in the future…	I am more likely to request a scan/another scan (e.g., x‐ray or MRI) I am neither more nor less likely to request a scan/another scan I am more likely to question the need for a scan/another scan I am less likely to request a scan/another scan (e.g., x‐ray or MRI)

9	Medical procedures ‘Having watched the webinar(s), if given the option in the future…’	I am more likely to choose a medical procedure (such as surgery or injections) for managing my pain I am neither more or less likely to choose a medical procedure (such as surgery or injections) for managing my pain I am more likely to question the need for a medical procedure (such as surgery or injections) for managing my pain I am less likely to choose a medical procedure (such as surgery or injections) for managing my pain

10	Medication Do you take any opioid‐based medication^∗^ for your pain? ^∗^Opioid‐based medications include codeine, dihydrocodeine, tramadol, morphine, fentanyl, oxycodone, buprenorphine and diamorphine	Yes No

11	Medication ‘Having watched the webinar(s)…’	I am more likely to take opioid‐based medication for my pain in the future I am neither more nor less likely to take opioid‐based medication for my pain in the future I am less likely to take opioid‐based medication for my pain in the future

12	Expectations ‘Having watched the webinar(s)…’	I am neither more or less hopeful about my future I am more hopeful about my future I am less hopeful about my future

13	Talking about your pain ‘Having watched the webinar(s)…’ ^∗^You can select multiple options	I feel more confident to discuss my pain and my pain management options with my employer I do not feel more or less confident to discuss my pain or my pain management with others I feel more confident to discuss my pain and my pain management options with a healthcare professional I feel more confident to discuss my pain and my pain management options with family and friends I feel less confident to discuss my pain and my pain management options with others

14	Would you recommend our webinars? Select as many as you feel is appropriate ^∗^You can select multiple options	I would recommend the webinar(s) to people living with chronic pain I would recommend the webinar(s) to anyone with loved ones living with chronic pain I would recommend the webinar(s) to employers of people living with chronic pain I would recommend the webinar(s) to healthcare professionals I would recommend the webinar(s) to everyone I wouldn’t recommend the webinar(s)

15	What did you find most useful about the webinar(s)?	Free text (Quals)

16	Has attending the webinar(s) changed what you expect from, or how you feel about, future healthcare appointments regarding your pain?	Free text (Quals)

17	What would you say to other people living with pain who may be considering attending one of our webinars?	Free text (Quals)

### 2.4. Analysis

Quantitative data were processed and presented as descriptive statistics. Data were aggregated across webinars and included all participants who completed the surveys irrespective of the nature of their waiting list. There was no subgroup analysis according to specific waiting lists. The initial stages of reflexive thematic analysis (data familiarisation, coding and generating initial themes) from open‐ended questionnaires were undertaken by M.J. [29]. Themes were then developed, reviewed, refined and defined with the assistance of V.V. and R.C.G. The final stage was collaborative and reflexive, undertaken by M.J. and R.C.G., to produce a coherent summary of the meaning and essence of experiences of PSE grounded in the words of the participants. Minority views were also identified and accounted for in creating themes.

## 3. Results

### 3.1. Participant Characteristics

The 114 participants with persistent pain in this study were on a waiting list for a variety of different specialities: Orthopaedics *n* = 33 (29.0%), Rheumatology *n* = 6 (5.3%), Pain Management Services/Pain Clinic *n* = 55 (48.2%), Physiotherapy/Musculoskeletal *n* = 13 (11.4%), Other (including Neurology, Urology and Gynaecology) *n* = 7 (6.1%).

The numbers of waiting list participants at each of the three webinars are documented in Table 3 below.

**Table 3:** Number of participants at each webinar.

Talk No.	Number of waiting list participants who completed the postevent evaluation
One—‘Rethinking Pain: New Understanding & New Possibilities’	101
Two—‘Why Everything Matters When it Comes to Pain’	40
Three—‘Flippin’ Everything You Thought You Knew About Arthritis’	25
One and two	18
One and three	2
Two and three	2
One, two and three	14

### 3.2. Responses to Quantitative Questions

A large majority of participants indicated that after the PSE they felt more ‘hopeful’ about their future with persistent pain. The majority of participants indicated that they intended to increase their activity level (71%), though 18% wanted guidance on how to do this. Of those using opioids, 51% thought that they were more likely to seek help to reduce opioid use. Further, 18% thought that they were less likely to request a scan, with 31% reporting they were more likely to question the need for a scan. With respect to views of the necessity of medical procedures, such as surgery or injections, 29% said that they were more likely to question the need for a medical procedure to manage pain, and 16% said they were less likely to choose a medical procedure. See Table 4 for responses on these topics with a categorised breakdown of responses.

**Table 4:** Participant hopefulness and intentions post‐PSE.

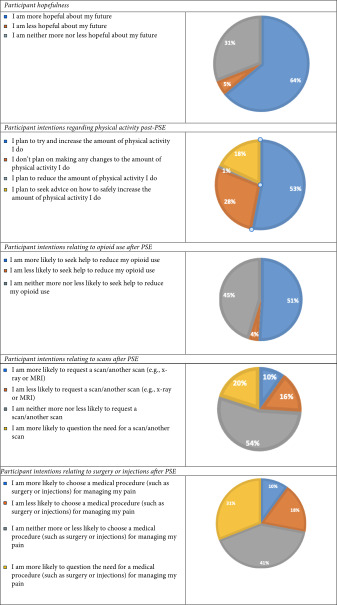

Overall participants felt that they would be markedly more confident to discuss their pain with others, with 64% saying that this applied to their conversations with healthcare professionals, and 48% felt more confident to discuss their pain with friends and family. Thirteen percent of participants considered that they would be more likely to discuss their pain with employers, and 25% felt there was no change in their likelihood of discussing their pain with others.

A total of 80% of participants reported they would recommend the webinars to people with persistent pain, and 65% reported they would recommend webinars to loved ones with persistent pain, whilst 59% recommended the webinars to ‘everyone’. Only 7% of the participants would not recommend the webinars to anyone.

### 3.3. Qualitative Results

The main and dominant themes were that the webinars left many people hopeful and that they intended to make changes to aspects of their lives affected by pain. A minority view expressed by four individuals was that the material lacked relevance.

### 3.4. Hope

The majority of participants reported that they felt more hopeful that they were not alone in having persistent pain. They appreciated hearing from passionate speakers and people with lived experience of pain.

‘Hope and ambition from those in the know, until I too can get there.’ (P 45)

### 3.5. Empowerment

Participants largely found the material interesting and up to date, and they valued expert opinion or reiteration of it. They also found the signposting to resources helpful with some describing the information as ‘lifechanging’.

*‘Hearing people who are/have been living with pain share their experience. Having the new science reiterated in different and clear ways. Hearing from people who are working with this science, so knowing that it’s up to date. Hearing about different techniques and resources and strategies for living with and reducing pain.’* (P 55 when asked ‘What did you find most useful about the webinars?’)

Empowerment took the form of requests individuals intended to make at future appointments. Whether they actually did or not is not, of course, assessed within this study. Ultimately, participants reported that they did not intend to seek biomedical intervention as much; they intended instead to seek interventions consistent with clinician‐supported biopsychosocial management or self‐management supported by the signposting provided in the webinars, such as websites for further information and healthcare professionals. They would also willingly recommend the webinars to others, to whom they would advise that they should keep an open mind.

Minority views included fears that not all healthcare professionals have the same biopsychosocial understanding of pain. One individual expressed feeling ‘depressed’ after the webinars, whilst one individual expressed dismay that others did not have access to the information they received in the webinars.

*‘I will strongly advise to attend the webinars as I believe that most people living with pain will find the whole webinar or parts of it very helpful.’* (P7)

### 3.6. Lack of Relevance

There was a minority perspective that some participants did not find the material relevant to them. In one case there was a very strongly expressed preference for passive management of pain. One of the participants, whilst seeing the benefit of the material, expressed despair because though they understood the messaging, they still had pain.

*‘Nothing, it did not include anything of practical use to me.’* (P 17)

## 4. Discussion

This is the first study to explore the perceptions of people with persistent pain of receiving PSE online, en masse, whilst on an NHS waiting list. Promising outcomes relating to increased hopefulness, intended positive behaviour change and perceived quality of the information (i.e., would recommend the webinars to others) provide preliminary support for the intervention.

A key finding of this study is that en masse delivery of PSE increased hopefulness in the majority of waiting list participants. Hope is defined as a nuanced, cognitive process for change that involves concepts such as goal‐setting, agency and cognitive restructuring [34]. In past work evaluating those who have benefitted from PSE, concepts that were deemed valuable to improvement were typically those that increased hopefulness for change and improvement [35]. Overall, hope and optimism have been closely linked to a reduced negative effect of chronic conditions on clinical outcomes, with greater hope/optimism associated with improved symptoms and daily functioning [36, 37].

Indeed, it is important to consider here that a small percentage (5%) of participants reported that they were less hopeful following PSE, with qualitative data highlighting that a few of these participants were angry and found the PSE messaging off‐putting, or they felt despair as they felt there was no cure for their pain. Such negative experiences are important to consider during en masse PSE campaigns and may speak to the need for individual follow‐up for the small proportion in which the messages do not land.

Data suggest that active interventions are more effective than passive management strategies for persistent pain [38]. Following the PSE webinars, our data showed greater behavioural intention shifts from passive, low‐value interventions to an inclination to follow evidence‐based, active approaches to self‐management of pain (e.g., reduce opioids, reduce or question scans and surgery and increase physical activity levels). Such findings are promising, though claims of cause and effect cannot be stated, showing intended behavioural shifts in line with the educational content of the webinars and evidence‐based guidelines [20, 38, 39].

Despite the lack of individualisation (as per en masse delivery), the PSE‐based webinars were considered credible by participants. Credible and persuasive information is a key ingredient for behaviour change, and in response to the questions about the value of the webinars, 80% of participants felt that the webinars were valuable. Furthermore, 59% felt that everyone would benefit from the PSE, and they supported continued provision of these relatively inexpensive waiting list interventions.

Finally, en masse PSE webinars appeared to influence patient empowerment in the moment, after the webinar. This may or may not have been borne out when empowerment was put to the test in communications with others, for example, healthcare professionals. Participants indicated that they felt more confident to speak to healthcare professionals about their pain in a more informed way, including asking for/seeking evidence‐based management. This suggests that the webinars contributed positively to some aspects of participants’ health literacy. Health literacy is defined as the ability to access, understand, appraise and use information and services in ways that promote and maintain good health and well‐being [40]. Clearly not all of these aspects of health literacy following PSE were investigated. However, the qualitative data revealed that there was a fear that not all healthcare professionals were skilled in evidence‐based pain management such as that presented in the webinars. Thus, while the PSE intervention may be successful in shifting knowledge and beliefs, the opportunity to practise the knowledge (which is a key part of behaviour change [32]) may be hampered by external forces such as the stance of outdated healthcare professional views.

### 4.1. Limitations

As this is a single‐group, observational study with only one measurement time point, no claims of cause and effect can be made. While the questions were worded to capture change in attitudes/beliefs/intended behaviours following the webinars, using both a pre‐ and postintervention survey would have allowed capture of the magnitude of absolute change. Additionally, lack of longer‐term follow‐up, paired with use of self‐report measures, means that it is unknown if the en masse PSE intervention resulted in lasting and/or real‐world behaviour change. Future studies should investigate the long‐term effects of these educational webinars, including barriers and facilitators to effecting change. Additionally, use of self‐reported, intended behaviour change immediately after the webinars raises the possibility that we did not capture change that may have occurred only after participants had time to reflect upon and use their new understanding in their everyday lives. The three webinars were evaluated as one entity, and there were no between‐webinar analyses. Future studies could assess if there was any difference in perception and perceived value of each webinar. Finally, an important limitation was that demographic information about participants was not recorded.

## 5. Conclusion

Participants on NHS waiting lists who had persistent pain found webinars that provided up‐to‐date PSE content to be helpful in shifting their understanding of pain. They reported the webinars were helpful in changing their approach to pain towards self‐management and generated feelings of hope. Participants intended to take a more active approach to managing their pain, including increased activity, reduced opioid use and strategies such as talking to others about their pain. Overall, participants intended to shift their self‐management of pain in line with evidence‐based recommendations. Appropriately powered RCTs with longer‐term follow‐up and objective measures of behaviour change (e.g., reduced surgery rates, increased physical activity levels) are warranted to robustly investigate the effectiveness of en masse PSE for patients awaiting care.

## Disclosure

This study was presented in poster format at the 14^th^ Congress of the European Pain Federation (EFIC) in Lyon, France, in April 2025. The conference abstract can be found at https://onlinelibrary.wiley.com/doi/10.1002/ejp.70133.

## Conflicts of Interest

Stanton T. R. received payment for lectures on pain and rehabilitation as well as payment for travel and accommodation costs from various societies and organisations. She also receives author royalties for a book she has written on osteoarthritis reconceptualisation and rehabilitation. Ryan C. G. and Mankelow J. create and deliver pain education material for Flippin’ Pain, a public health campaign in the United Kingdom, funded by Cora Health. Their employing university receives consultation fees for their work on the campaign, but Ryan C. G. and Mankelow J. receive no personal income from the work.

## Funding

No funding was received for this manuscript.

## Data Availability

The data that support the findings of this study are available on request from the corresponding author. The data are not publicly available due to privacy or ethical restrictions.
